# CLiP, catheter and line position dataset

**DOI:** 10.1038/s41597-021-01066-8

**Published:** 2021-10-28

**Authors:** Jennifer S. N. Tang, Jarrel C. Y. Seah, Adil Zia, Jay Gajera, Richard N. Schlegel, Aaron J. N. Wong, Dayu Gai, Shu Su, Tony Bose, Marcus L. Kok, Alex Jarema, George N. Harisis, Chris-Tin Cheng, Helen Kavnoudias, Wayland Wang, Anouk Stein, George Shih, Frank Gaillard, Andrew Dixon, Meng Law

**Affiliations:** 1 Department of Radiology, The Royal Melbourne Hospital, Melbourne, Victoria Australia; 2 Department of Radiology, Alfred Health, Melbourne, Victoria Australia; 3 Alfred Health, Melbourne, Victoria Australia; 4 Department of Radiology, Monash Health, Melbourne, Victoria Australia; 5 Barwon Imaging, Geelong, Victoria Australia; 6 The Royal Melbourne Hospital, Melbourne, Victoria Australia; 7 Eastern Health, Box Hill, Victoria Australia; 8 Department of Surgery, Monash School of Medicine, Nursing and Health Sciences, Clayton, Victoria Australia; 9 MD.ai, New York, New York USA; 10 Department of Radiology, Weill Cornell Medicine, New York, USA; 11 Faculty of Medicine, Dentistry and Health Sciences at the University of Melbourne, Melbourne, Victoria Australia; 12 Department of Electrical and Computer Systems Engineering, Monash University, Clayton, Victoria Australia; 13 Department of Neuroscience, Monash School of Medicine, Nursing and Health Sciences, Clayton, Victoria Australia; 14 Departments of Neurological Surgery and Biomedical Engineering, University of Southern California, Los Angeles, USA

**Keywords:** Radiography, Medical imaging

## Abstract

Correct catheter position is crucial to ensuring appropriate function of the catheter and avoid complications. This paper describes a dataset consisting of 50,612 image level and 17,999 manually labelled annotations from 30,083 chest radiographs from the publicly available NIH ChestXRay14 dataset with manually annotated and segmented endotracheal tubes (ETT), nasoenteric tubes (NET) and central venous catheters (CVCs).

## Abstract (summary)

**Table:** 

Measurement(s)	Catheter Device
Technology Type(s)	Annotation
Sample Characteristic - Organism	Homo sapiens

Machine-accessible metadata file describing the reported data: 10.6084/m9.figshare.15113043

## Background & Summary

Radiographs are considered the gold standard for confirmation of line and tube position^1^. Non-radiologists, such as emergency physicians and intensivists, often interpret the radiographs when they are taken and identify clearly malpositioned catheters, however, suboptimally positioned catheters may be missed. Whilst these suboptimally positioned catheters are often picked up subsequently by radiologists, there may be a time delay between when the radiograph is taken and the report issued. Early recognition and repositioning are important to prevent further complications associated with tube and line malposition.

With the increasing application of machine learning, and in particular deep learning in medicine and radiology, there is an increasing need for publicly available clinically relevant labeled datasets to allow side-by-side evaluation of algorithms.

There are several publicly available large scale chest radiograph datasets currently available including the MIMIC-CXR dataset and the NIH ChestXray14 dataset^2,3^. The MIMIC-CXR set contains 227,835 imaging studies for 65,379 patients with associated radiology reports. The NIH ChestXray14 dataset was released in 2017 and comprises over 112,120 frontal radiographs from 30,805 patients. The dataset was labeled using natural language processing applied to the original free-text reports which involved matching keywords to certain pathologies. The dataset was initially released with eight different classes and was then expanded to fourteen classes. A subset of this dataset contains catheters, however, currently, there are no publicly available labels that describe the presence or position of these catheters.

A limitation of developing labels from reports is that there is a heavy reliance on the report itself which often has incomplete descriptions of the images, it has been demonstrated that better correlation with the visual content of images can be found in manually labeled datasets, however this is significantly more time intensive^4^.

Additionally, whilst these publicly available datasets are often produced by a large team of researchers and clinicians, there is often a disconnect between these individuals and those that use the dataset, often computer engineers and computational scientists. Although the limitations of each dataset are frequently described by their creators, the end-users of these datasets are sometimes non-medical and/or lacking specialty radiology knowledge and may therefore not fully appreciate the clinical significance of these limitations.

This paper describes the manual annotation of the endotracheal tubes (ETT), nasoenteric tubes (NET), and central venous catheters (CVCs) in the NIH ChestXRay14 dataset into clinically relevant categories. The final dataset contains 30,083 chest radiographs with image-level labels of which a subset of the chest radiographs have accompanying manually segmented labels.

## Methods

### Selection of radiographs

As the NIH ChestXray14 dataset comprises over 100,000 chest radiographs, many without catheters, relevant radiographs were selected by an algorithm that was developed on the MIMIC-CXR set.

A case-insensitive keyword search of the reports accompanying the MIMIC-CXR set was performed in order to create noisy labels for the deep learning algorithm. Table 1 demonstrates the search terms used in the ETT, NET, and CVC categories. A DenseNet algorithm was then used to train a deep learning model on the radiographs and accompanying reports to identify the presence or absence of the catheters. The algorithm identified approximately 45,000 relevant chest radiographs and prioritized them in order based on the probability of a catheter being in the image, as well as the probability of a borderline or abnormally positioned catheter being in the image. Selected images from the algorithm output were then manually reviewed and approximately 35,000 selected for annotation.

**Table 1:** Search terms used in the keyword search in the algorithm developed from the MIMIC-CXR dataset.

Endotracheal Tube	Nasoenteric Tube	Central Venous Catheter
ETT	NET	Central line
Endotracheal	NGT	Jugular line
Intubate	nasoenteric	Subclavian line
ET tube	Dobhoff	Central venous
	Dobbhoff	CVC
	Nasoenteric	Lines and tubes
	NG tube	Hickman
	Nasojejunal	IJ Line
	Nasoduodenal	Port

### Ethical statement

The datasets used in the development of this dataset include MIMIC-CXR and NIH ChestXray14 both of which are databases of chest radiographs which have been previously de-identified. As both datasets are publicly available IRB approval is waived and in development of this dataset, we complied with all relevant ethical regulations and NIH ChestXray14 and MIMIC-CXR data usage guidelines on the relevant database websites https://physionet.org/content/mimic-cxr/2.0.0/ and https://nihcc.app.box.com/v/ChestXray-NIHCC^3,5^.

### Exclusion criteria

All radiographs from individuals less than 10 years old were removed given the differences in paediatric lines, with optimal line position on chest radiograph differing to that in the adult population.

Furthermore, diagnostically inadequate studies were removed including lateral projections, abdominal radiographs, and radiographs with poor exposure or significant rotation.

### Annotation of data

The included chest radiographs were uploaded to the browser-based annotation interface, MD.ai^6^. No image processing was performed on the chest radiographs prior to upload. The labelers used personal computers to label the data online with the interface allowing individuals to window, pan, and zoom images. There were a total of 43 labelers including radiologists, radiology trainees and hospital medical officers. Each labeler underwent a standardized training session and were provided with a document of definitions to ensure consistency of labels.

Image level labels were applied to all radiographs, with segmented labels also applied to a subset of the radiographs, categorised into three clinically relevant categories: normal, borderline, and abnormal. The normal category included lines that were appropriately positioned and did not require repositioning. The borderline category included lines that would ideally require some repositioning but would in most cases still function adequately in their current position. The abnormal category included lines that required immediate repositioning.

These definitions, where possible, used anatomical references as guides and scales as many of the chest radiographs in the NIH ChestXray14 dataset are mobile frontal ICU radiographs where measurements are unreliable due to variable patient to x-ray tube distance and geometrical alignment, which results in distortion and magnification of the image.

Segmentation of NETs and ETTs were performed with the line of interest through the centre of the tube only within the segment internal to the patient. The CVCs, in contrast, were segmented in their entirety from their hub to their tip, as it can be difficult to differentiate the exact skin-entry location.

### Definitions

#### Anatomical references

To allow for consistency between labelers, a set of definitions were developed for each catheter type with the use of anatomical references to guide position as well as to develop a scale within the radiograph.

##### Carina.

The carina was defined as the point where the inferior wall of the left and right main bronchi intersect. This anatomical reference was used in the ETT definition.

##### Aortic Arch.

The aortic arch was defined as the superior margin of the aortic knob (the prominent shadow of the aortic arch). This anatomical reference was used in the ETT definition as well as the CVC definition to assist in defining the upper margin of the superior vena cava.

##### Cavo-Atrial junction.

The cavo-atrial junction was defined as the intersection between the right heart border and the inferior margin of the bronchus intermedius^7^. When the bronchus intermedius was not clearly identified, labelers were advised to use the change in curvature from the SVC contour and the right heart border as a guide. This anatomical reference was used in the central line definition.

##### Gastro-esophageal junction.

The gastro-esophageal junction was defined as the medial border of the left hemidiaphragm. In cases where there was obscuration of the left hemidiaphragm from collapse or consolidation, labelers were asked to make the best estimate based on the position of the right hemidiaphragm and the inferior aspect of the cardiac shadow. This anatomical reference was used in the NET definition.

Ribs and ribs spaces were used to develop scales within the radiographs which were used in the ETT and NET definition. Two definitions were developed for the purposes of labeling this dataset called the “Two posterior ribs” and “Four posterior ribs” definitions to assist in providing a scale for the labelers. The rib margins were used as they are better defined than the centre of the rib or the rib space.

##### Two posterior ribs.

**Fig. 1:**
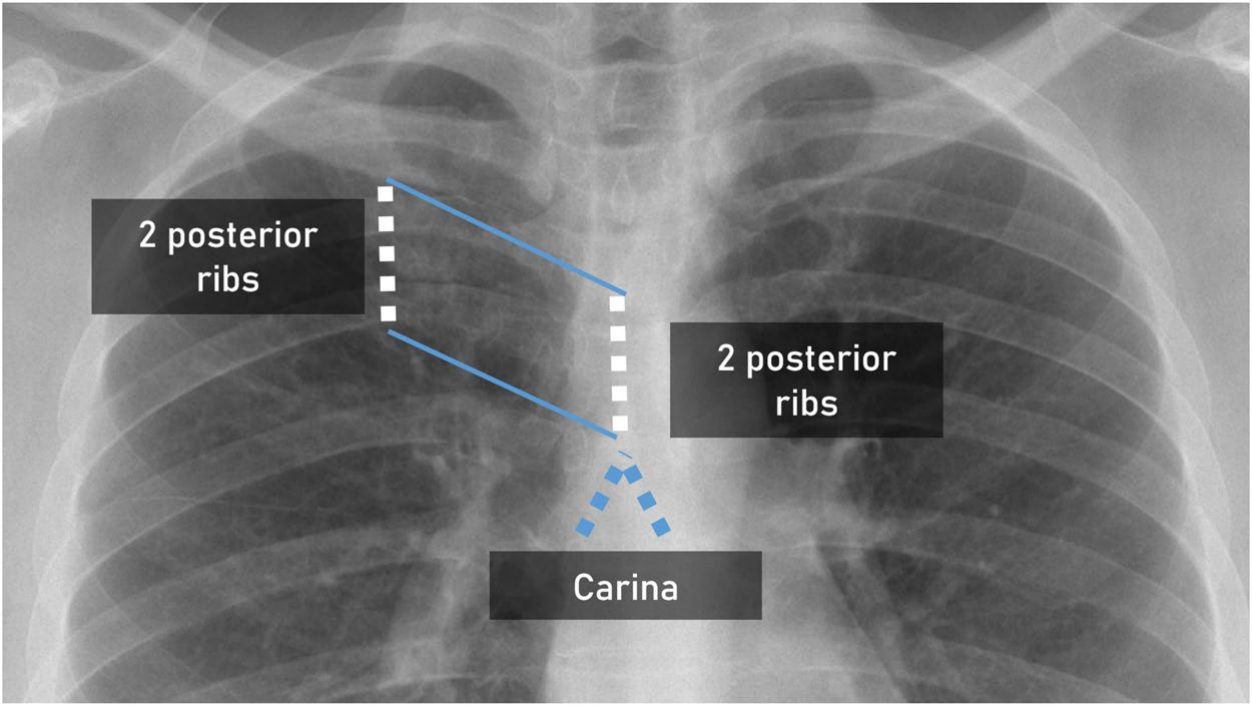
Illustration of the “Two posterior ribs” definition.

The “Two posterior ribs” definition was defined as the distance between the superior margin of one rib to the inferior margin of the rib below in the upper thoracic region as seen in Fig. 1. This was intended to provide a scale for the ETT definition with the “Two posterior ribs” equating to approximately 3.5 cm.

This was based on known measurements of human ribs in the upper thoracic region. The human rib measures approximately 1.5 cm with the posterior intercostal space in the upper thoracic region measuring approximately 0.5–0.6 cm^8,9^. Therefore the 2 posterior ribs and the intervening intercostal space in the upper thoracic region measures approximately 3.5 cm.

##### Four posterior ribs.

**Fig. 2:**
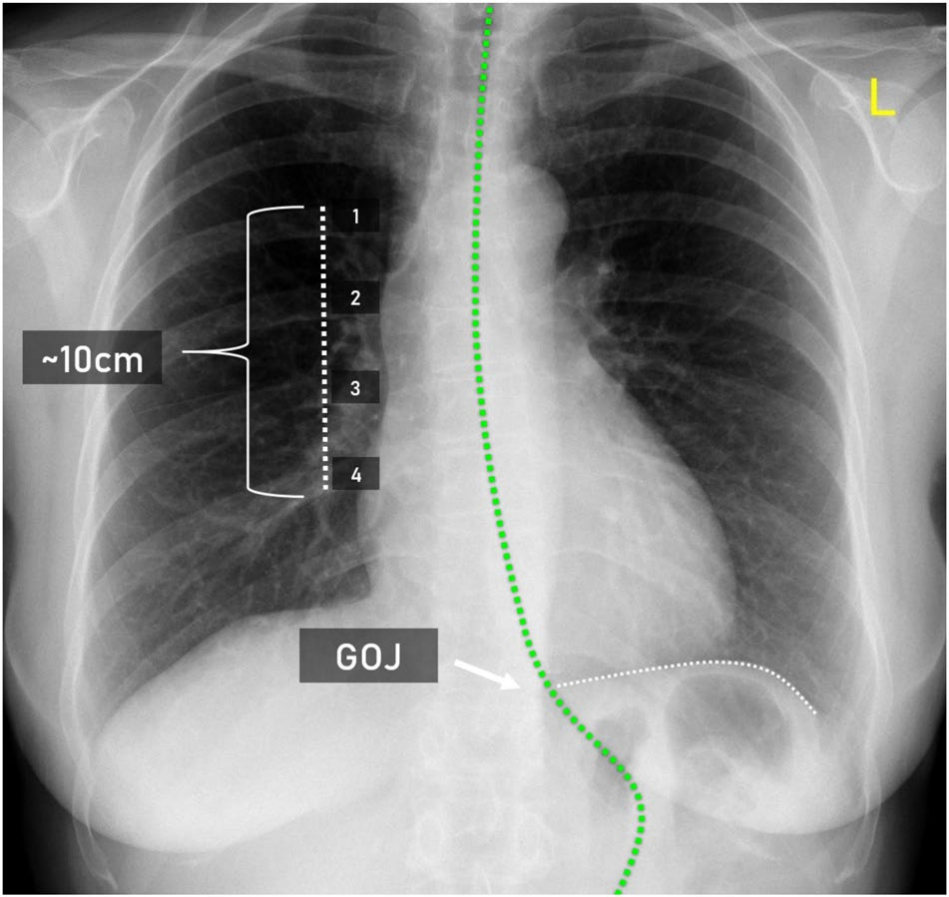
Illustration of the scale that was used to assist in approximating 10 cm below the gastro-esophageal junction.

The “Four posterior ribs” was defined as the distance from the superior border of one rib to the inferior border of the third rib below in the lower thoracic region, making a total of 4 posterior ribs and 3 rib spaces (Fig. 2). This was intended to provide a scale for the NET definition with “Four posterior ribs” equating to approximately 10 cm. Unlike the “Two posterior ribs” definition where ribs in the upper thoracic region were used, this definition involved the use of the lower thoracic paraspinal ribs.

This definition was based on known measurements of human ribs in the lower thoracic region. The intercostal space width and rib width of the lower posterior ribs in humans are approximately 15.1 ± 2.3 mm in width and the intercostal space approximately 14.5 ± 3.6 mm in width^9^. Therefore, a total of 4 posterior ribs and 3 rib spaces equals approximately 10 cm in total.

#### Endotracheal tubes (ETT)

Malpositioning of endotracheal tubes (ETT) in adult patients intubated outside the operating room is seen in up to 25% of cases^10,11^. Early recognition and repositioning is important to avoid complications such as atelectasis, pneumothoraces, and inadequate ventilation. When the head and neck are in a neutral position the tip of the ETT should ideally be 3–7 cm above the carina^13^. This allows for the tube to remain in the trachea during neck flexion, which results in the tip descending, and extension, which results in the tube ascending^12,13^^,^.

**Fig. 3:**
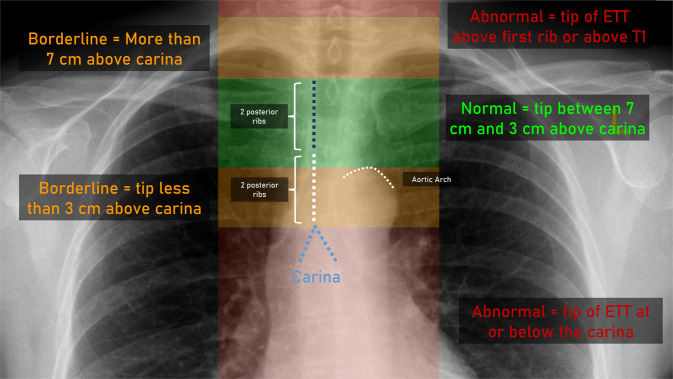
Illustration of the ETT definition.

Tracheostomies were also included in the ETT definition. The rationale for including tracheostomies was that they would serve the same function (ventilation) as endotracheal tubes. To reduce the total number of labels, the tracheostomies were not labeled in a separate category. An illustration of the ETT definition is demonstrated in Fig. 3.

##### Normal.

An ETT was categorised as normal if the ETT tip was above the upper margin of the aortic arch. The superior aspect of the aortic arch is located approximately 3–5 cm above the carina and therefore ETT position at the same level or slightly above is acceptable. As the aortic arch was not always readily visible in some radiographs, the posterior ribs were also used as a guide with an ETT more than “Two posterior ribs” above the carina considered to be normal.

##### Borderline.

A borderline ETT was considered in two scenarios, where the ETT was borderline low or borderline high. If the ETT tip was below the aortic arch this was considered borderline low. When the ETT tip was less than “Two posterior ribs” above the carina this was also considered borderline low. If the ETT tip was located higher than two times “Two posterior ribs” above the carina which measures approximately 7 cm, this was considered borderline high.

##### Abnormal.

An abnormal ETT was considered in two scenarios, where the tip of the ETT was at or below the carina and if the ETT tip was at the level of T1 or above.

##### Tracheostomy Tubes.

Readjustment of tracheostomy tubes is reliant on clinical assessment and post-tracheostomy tube placement chest radiographs have been shown to not alter patient management^14^, with some even deeming them unnecessary^15^. Therefore, to maintain the clinical relevance of the definitions, all tracheostomy tubes were labeled in the “ETT normal” category.

#### Nasoenteric tubes (NET)

The chest radiograph remains the gold standard to verify the correct placement of nasoenteric tube position^14^. Nasoenteric tube complications can be divided into tracheobronchopleural, intravascular, enteral, and intracranial complications^14^. In an intensive care environment, approximately 2% of NETs have been shown to be inserted into the tracheopulmonary system with 0.3% directly contributing to patient death^16,17^.

As nasoenteric tubes often have one or more side holes that are variably located up to 10 cm from the tip but not always visible on a radiograph, the nasoenteric tube definition needs to account for their presence without relying upon them as a landmark^15^. Therefore, the tip of the NET when included in the radiograph, or the lowest point of the NET visible on the radiograph when the tip was outside the field of view, was used. The NET definition comprises four categories, normal, borderline, abnormal, and incompletely imaged.

##### Normal.

The position of a NET was considered normal when the tip of the NET was at least 10 cm from the gastro-esophageal junction, defined as the medial border of the left hemidiaphragm. The “Four posterior ribs” definition was used as a scale in the radiographs to approximately 10 cm.

##### Borderline.

Borderline position was considered when the tip of the NET was beyond the gastro-esophageal junction but appeared to be less than 10 cm from the gastro-esophageal junction. The NET was also considered borderline if the line was kinked within the stomach.

##### Abnormal.

Any NET within the tracheobronchopulmonary system or within the esophagus above the gastro-esophageal junction was considered abnormal. Additionally, if the NET was coiled or looped anywhere along its path above the gastro-esophageal junction or with its tip in the esophagus this was considered abnormal.

##### Incompletely imaged.

This category was included for the examinations where there was insufficient tube within the radiograph to make a decision as to whether it was normal or not. If the NET tip was outside the field of the radiograph but there was >10 cm within the radiograph, this was considered sufficient information, and the NET was considered normal.

#### Central venous catheters (CVC)

All central venous catheters and peripherally inserted central catheters (PICCs) were included in this definition. The use of CVC in the definitions will encompass both of these. Central venous catheter malposition occurs in approximately 7% of cases^18^. High position of the catheter is associated with increased risk of thrombosis, whilst low position in the right atrium can be associated with dysrhythmias^7^. There is no clear consensus for ideal central venous catheter position, with differences in position recommended for left versus right sided approach CVCs. Overall, the position within the SVC at the level of the cavo-atrial junction is considered adequate^19^. As it has been shown that an angle of the CVC tip to the vessel wall of greater than 40 degrees is more likely to lead to vessel wall perforation, angle of the tip is also taken into account in the development of the definitions^20^.

The tip of Swan Ganz Catheters were not assessed in this dataset, but the presence or absence of a Swan Ganz Catheter was documented. A presumption was made that all Swan Ganz Catheters would be inserted through a CVC sheath and given it is sometimes difficult to delineate between where the CVC sheath stops, all Swan Ganz Catheters were automatically labeled with a “CVC normal”.

##### Normal.

CVCs were considered to have a normal position if their tip projected over the SVC, below the upper margin of the aortic arch and above the cavoatrial junction. The catheters also had to make an angle of less than 45 degrees to the vessel wall. An angle of 45 degrees instead of 40 degrees was used as it was easier to estimate.

##### Borderline.

A borderline CVC was a line that was along the expected path, including in the arm, but either positioned proximal to the SVC, within the SVC but with an angle to the vessel wall of >45 degrees or below the cavoatrial junction but with the tip still remaining in the upper 1/3 of the right atrium.

##### Abnormal.

An abnormal CVC was defined as the tip of the central line below the upper 1/3 of the right atrium, if the line was not following the expected path or if the line, or was coiled or kinked anywhere along its expected internal path. Any central line with an atypical position, such as with the tip in the azygos vein or internal thoracic vein, or into the aorta or extravascular were also considered as abnormal.

### Resulting dataset

The dataset includes 30,083 chest radiographs from 3791 patients with a median age of 49. From the 30,083 there are 50,612 image level annotations and 17,999 manually segmented annotations. Approximately 30% of the radiographs were double-labeled and 10% were triple-labeled. A breakdown of the numbers of each type of catheter is outlined in Table 2.

**Table 2:** Count of each catheter type in the dataset.

Catheter Type	Count
ETT Abnormal	79
ETT Borderline	1138
ETT Normal	7240
NET Abnormal	267
NET Borderline	530
NET Incompletely Imaged	2748
NET Normal	4799
CVC Abnormal	3198
CVC Borderline	8460
CVC Normal	21323
Swan Ganz Catheter Present	830

#### Assumptions

Catheters included in the CVC category included ports, Hickmann’s catheters, central lines, and PICCs. As the annotations were performed in a non-clinical setting, no clinical information about the use of the tubes was provided to labelers and for the purposes of this dataset, the definitions were applied to all catheters regardless of type or use.

#### Limitations

A limitation is that a lot of chest radiographs were rotated or have non-standard positioning which made strict application of the definitions difficult and labelers were instructed to use their clinical judgment in such scenarios, which could lead to an increased disparity between annotators.

An inherent limitation of projectional radiography is that lengths can be underestimated as these are two dimensional representations of three dimensional objects. In practice, this means that the definitions adopted in this paper are conservative, and that deep learning models trained off this dataset are likely to have a higher sensitivity.

Catheter position on radiographs at baseline is associated with variability given there is no single definition for optimal line position. General variation in perceptual skills among observers and experience also contributes to variability. This was mitigated by providing a clear set of definitions for the labelers, training sessions, and constant auditing throughout the labeling process. Furthermore, the use of double and triple labels, with labelers blinded to the other labels, also helps to improve the consistency of the labels.

## Data Records

The labels and each of the corresponding radiographs are available on Kaggle, which is a global data science and machine learning competition platform^21^. Access requires user registration and acceptance of data licensing. Classification labels are provided as a CSV in a single file. Segmentation labels are provided as lists of coordinates with the keys corresponding to the type and position of that line. All images were upscaled to original resolution before conversion to JPEG and uploaded into the Kaggle repository.

## Technical Validation

To ensure consistency of the labels and concordance with the definitions each labeler underwent a 30-minute training session and auditing of the labels throughout the labeling process was performed. Feedback was provided to the labeler if the labels were not consistent with the definitions.

A consensus algorithm was developed on a modified Dawid Skene model to determine the reliability of the labeler in each of the categories. The consensus algorithm was applied to all double and triple labeled radiographs.

5100 consensus labels were randomly reviewed by experienced labellers, with approximately 15% discrepancies demonstrated. These labels were then used as the “gold standard” label and used to train the Dawid Skene model again to improve its output for the remaining double and triple labels. Labellers with high accuracy rates as per the consensus algorithm were subsequently allowed to label radiographs individually. Additional labels for these radiographs were considered unnecessary as error rates for these labellers were low.

## Usage Notes

The dataset has been utilized in a Kaggle competition where multiple publicly accessible notebooks demonstrate usage of this data to train machine learning models. We hope that users will continue to contribute code and updates to this dataset to accelerate research in this field.

## Data Availability

1. Data preparation DenseNet model applied to the MIMIC-CXR dataset which was subsequently used to extract chest radiographs with catheters and lines from the NIH Chest X-ray dataset. The model been made available https://github.com/jarrelscy/cxr-lines-tube-model, which interfaces with the MD.ai interface to perform inference and identify chest radiographs with catheters and lines in the NIH Chest X-ray dataset. 2. Technical validation Preprocessing script describing used in determining final labels for the chest radiographs. This code has been made available at https://github.com/jsntang/ranzcr_kaggle_preprocessing however the raw data export is not released as this contains identifying personal information of the labellers.
